# Highly Variable Clinical Pictures in Dogs Naturally Infected with *Angiostrongylus vasorum*

**DOI:** 10.3390/pathogens10111372

**Published:** 2021-10-23

**Authors:** Mariasole Colombo, Donato Traversa, Eleonora Grillotti, Carlo Pezzuto, Cesare De Tommaso, Fabrizio Pampurini, Roland Schaper, Jason Drake, Paolo Emidio Crisi, Ilaria Russi, Marco Ripamonti, Angela Di Cesare

**Affiliations:** 1 Faculty of Veterinary Medicine, University of Teramo, 64100 Teramo, Italy; mcolombo@unite.it (M.C.); eleonoragrillotti@gmail.com (E.G.); pecrisi@unite.it (P.E.C.); ilariabak@gmail.com (I.R.); adicesare@unite.it (A.D.C.); 2 Ambulatorio Veterinario Reate, 02100 Rieti, Italy; 3 Ambulatorio Veterinario Pezzuto Carlo/Piano Noemi, 86010 Campobasso, Italy; pezzutoepiano@gmail.com; 4 Labforvet, 81100 Caserta, Italy; labforvetcaserta@gmail.com; 5 Elanco Animal Health, Indianapolis, IN 46240, USA; fabrizio.pampurini@elancoah.com (F.P.); roland.schaper@gmx.de (R.S.); jason.drake@elancoah.com (J.D.); marco.ripamonti@elancoah.com (M.R.)

**Keywords:** *Angiostrongylus vasorum*, angiostrongylosis, clinical signs, dogs

## Abstract

Canine angiostrongylosis by *Angiostrongylus vasorum* is increasingly reported in both enzootic and previously free areas. The complex pathogenesis of the disease makes the clinical workup challenging. Infected dogs show highly variable clinical pictures, characterized by subclinical to life-threatening general, cardio-respiratory, neurological and/or gastrointestinal signs. The present study reports the high variability of clinical pictures from 36 dogs across central and southern Italy that were naturally infected by *A. vasorum*. Of them, 23 (63.9%) presented at least one clinical sign, while 13 (36.1%) were subclinically infected and apparently healthy. Overall, 19 dogs (52.8%) showed cardiorespiratory signs, 14 (38.9%) had non-specific abnormalities, 2 (5.6%) presented coagulation disorders and 1 (2.8%) had a severe neurological condition. Importantly, four dogs presenting with clinical signs had neither cough nor dyspnea. These results underline that angiostrongylosis should be included in the differential diagnosis, even when dogs display only non-specific clinical signs. The proportion of apparently healthy dogs highlights the relevance of routine copromicroscopic and/or antigenic tests in enzootic areas to avoid the sudden onset of potentially life-threatening signs.

## 1. Introduction

The infection of companion animals with nematodes of the genus *Angiostrongylus* has recently gained attention in small animal clinical practices. While the role and importance of feline angiostrongylosis is yet to be understood [1,2,3], canine angiostrongylosis by *Angiostrongylus vasorum* is emerging as one of the most important parasitosis in canine medicine [4,5]. Adult stages of *A. vasorum* live in the pulmonary arteries and in the right heart of the definitive hosts, i.e., dogs, foxes and other animals [6,7,8]. After mating, *A. vasorum* females lay eggs that hatch and release first stage larvae (L1), which penetrate the alveolar/bronchial walls, reach the pharynx and are then swallowed and excreted with feces in the environment [9]. Dogs become infected ingesting either the intermediate (i.e., slugs and snails) or paratenic hosts (i.e., frogs and poultry) harboring the infective third stage larvae (L3) [7,10,11]. There is a recent hypothesis that dogs may acquire angiostrongylosis potentially via ingestion of infective L3 shed by slugs or snails onto vegetation in the environment [12].

The pathogenesis of the disease is complex and relies on different mechanisms [13,14,15] including (i) the presence of adults in the lungs with mechanic impairment of pulmonary arteries, (ii) lung inflammation as a response to eggs and migrating larvae, (iii) coagulation disorders, most probably due to hyperfibrinolysis and hypofibrinogenemia, as well as alterations in the coagulation cascade. Coagulation disorders cause hemorrhages in different body parts and when in the Central Nervous System (CNS), along with possible larval migrations, neurological clinical signs occur [16,17].

The clinical course is highly variable and unpredictable, and infected animals show varying pictures that can be subclinical, hyperacute/acute or chronic. Some dogs can be apparently healthy (Table 1) for a long period of time before an abrupt appearance of severe, and often fatal, clinical pictures [18,19,20,21].

Cardiopulmonary clinical signs, mainly cough and dyspnea, are most frequently reported [4,20,22]. Pulmonary hypertension and congestive right-sided heart failure can be fatal in more severe cases [23,24]. Bleeding disorders are frequent, and dogs may display epistaxis, hemoptysis, mucosal petechiae and ecchymosis [18,25,26]. Neurological signs include paresis/paralysis, ataxia and seizures [13,16,17]. Other clinical alterations are non-specific and include gastrointestinal signs, i.e., vomiting, diarrhea, anorexia, weight loss [20,27] and ocular lesions [28].

In recent years *A. vasorum* has expanded its geographic distribution, from the Iberian Peninsula [29,30] to Central and Northern Europe [31,32,33], across the Mediterranean basin [27,34] to countries of Eastern Europe [35,36]. Though awareness on this life-threatening parasitosis is increasing [9,27], practitioners are still faced with a challenging and unexpected disease where *A. vasorum* is neglected and underestimated.

A continuous update on clinical features in naturally infected dogs is crucial for a timely diagnosis and prompt appropriate treatments. Hence, this study was conducted to evaluate the occurrence of different clinical presentations in dogs naturally infected by *A. vasorum* in enzootic areas of Italy.

## 2. Results

**Figure 1:**
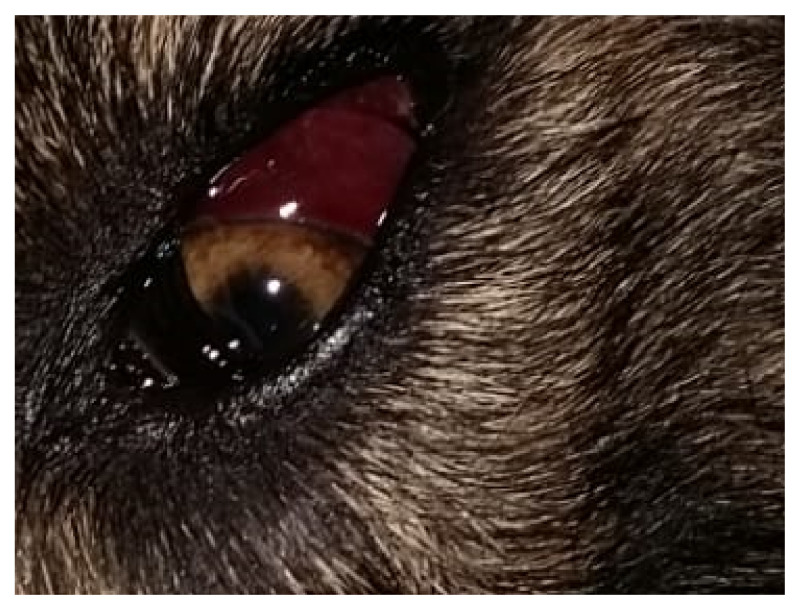
Dog infected with *Angiostrongylus vasorum* showing a spontaneous subconjunctival hemorrhage.

**Figure 2:**
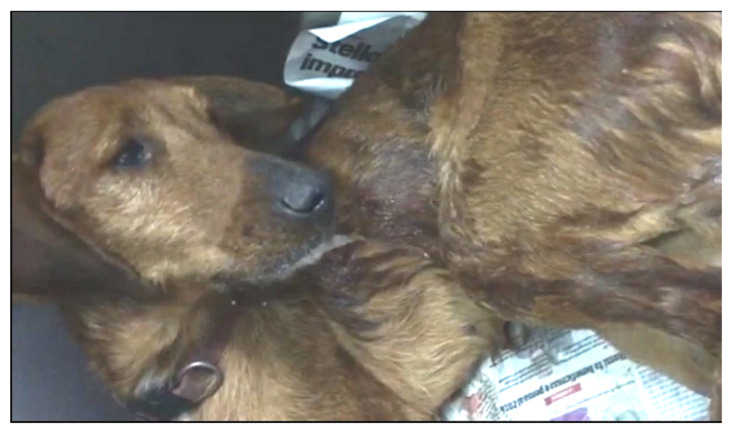
Dog infected with *Angiostrongylus vasorum* showing shivering and hypersalivation.

The age of the infected dogs ranged from 4 months to 14 years. Within this group, 20 dogs were female, 16 dogs were male, 34 dogs lived outdoors (e.g., gardens and boxes) and 2 prevalently indoors (with up to 2–3 walks/day). Of the 36 clinically examined animals, 23 (63.9%) showed clinical signs, while 13 (36.1%) were apparently healthy. In detail, 19 (52.8%) had cardio-respiratory disorders while 4 (11.1%) had neither cough nor dyspnea. Non-specific abnormalities, including weight loss, diarrhea, anorexia, lethargy, exercise intolerance and fever, were present in 14 (38.9%) dogs, while 2 (5.6%) presented coagulation alterations, i.e., hematochezia and spontaneous hemorrhages (Figure 1), respectively, and 1 (2.8%) had severe neurological signs (Figure 2).

**Figure 3:**
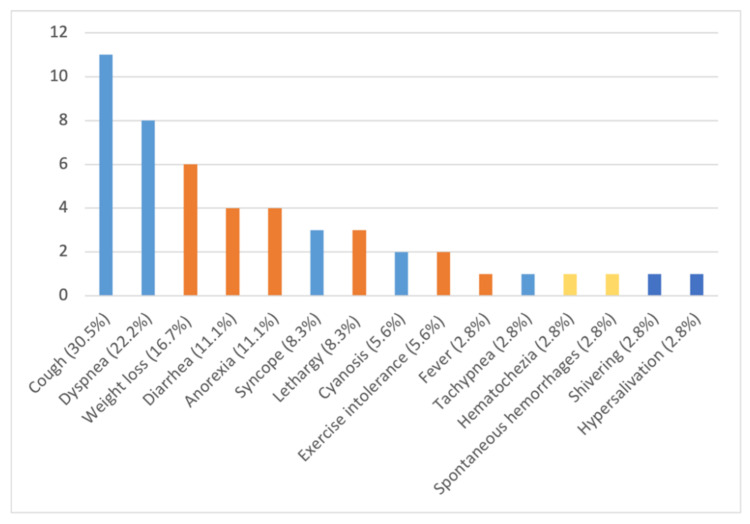
Number and percentage of clinical signs observed in 36 dogs naturally infected by *Angiostrongylus vasorum*: light blue: cardio-respiratory signs; orange: non-specific signs; yellow: coagulation disorders; blue: neurological signs.

**Figure 4:**
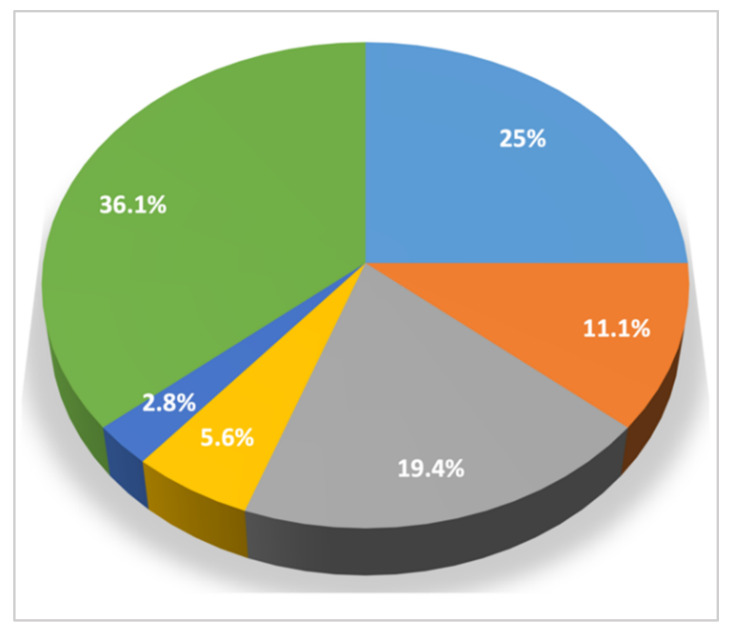
Clinical pictures and association between different categories of clinical signs observed in 36 dogs with angiostrongylosis: light blue: cardio-respiratory signs; orange: non-specific signs; grey: cardio-respiratory signs + non-specific signs; yellow: cardio-respiratory signs + non specific signs + coagulation disorders; blue: cardio-respiratory signs + non-specific signs + neurological signs; green: no clinical signs.

The dogs showed variable clinical pictures with a high number of combinations of signs (Figure 3 and Figure 4). In particular, 7 (19.4%) of the 36 infected dogs had a combination of cardio-respiratory and non-specific signs, 2 (5.6%) showed cardio-respiratory signs in association with non-specific signs and coagulation disorders, 1 (2.8%) was brought to visit for cardio-respiratory, non-specific and neurological (Figure 2) signs and 9 (25%) and 4 (11.1%) dogs had only cardio-respiratory and non-specific signs, respectively. The remaining 13 had no evident clinical sign during the clinical examination. Overall, 17 infected dogs (47.2%), 13 clinically healthy and 4 with other conditions did not show cardio-respiratory signs. Detailed information on the clinical signs observed are listed in Table 2 and are shown by the frequency of observation in Figure 3.

## 3. Discussion

These data show that canine angiostrongylosis occurs with multiple and highly variable clinical presentations, and that the absence of cardio-respiratory signs should not preclude the inclusion of *A. vasorum* as a differential diagnosis in enzootic areas. In fact, almost half of the infected dogs did not exhibit any pulmonary condition, and some had other clinical signs that were not specific for a definitive diagnosis of *A. vasorum* infection.

Nonetheless, cardio-respiratory signs, i.e., cough, dyspnea, syncope, cyanosis and tachypnea, were present in several dogs in the present study, with cough being the most frequent. This is consistent with the results of other case series, where cardio-respiratory signs were present in the 63–65% of infected dogs, with cough in 42–65% of the cases [4,18,22,26].

Coagulopathies are common in canine angiostrongylosis. Past studies showed that they can be present in 15–45% of the cases [4,18,22,26,37], and that sometimes they are primary alterations in dogs with angiostrongylosis [4,38]. In contrast, bleeding was present only in a small proportion of dogs in this study. Indeed, blood disorders, e.g., anemia or higher activated partial thromboplastin time, can sometimes be mild and occur with no clinically evident hemorrhages or hematomas [39]. A coagulation profile or diagnostic imaging procedure would have been useful to identify bleedings (e.g., internal hematomas or mild hemoabdomen/hemothorax) undetected during the clinical examination [40,41]. As this was not performed with these study dogs, the proportion of coagulopathies in this paper may be underestimated. For instance, neurological signs could be elicited by both blood disorders and aberrant larval migrations in the CNS [16,17]. Shivering and hypersalivation in the single dog with evident neurological conditions (Figure 2) could have been caused by hemorrhages in the CNS. This would increase the number of dogs with coagulation disorders due to *A. vasorum* in this study. Another cause of neurological damage is the aberrant migration of *A. vasorum* larvae. Regardless, neurological signs were minimally represented in accordance with previous studies, which reported a 4–16% occurrence [4,18,22,26,37].

The high number of dogs with non-specific signs, i.e., weight loss, diarrhea, anorexia, lethargy and exercise intolerance, is unsurprising. These signs are frequently recorded in canine angiostrongylosis, and sometimes they are the only clinically evident alterations [4,20,26]. Fever is an infrequent non-specific alteration in dogs with angiostrongylosis [4,22,26,42,43,44]. In general, pyrexia can be present in up to 17% of the cases, though sometimes it is the only evident clinical manifestation [18,45]. Accordingly, increased body temperature and lethargy were the only clinical findings in one dog in the present study, confirming that, in enzootic areas, *A. vasorum* infection should be suspected in dogs presenting with fever as the sole/main alteration.

Subclinical infections with *A. vasorum* are generally less frequently described in the literature when compared to clinically manifested angiostrongylosis [4,22,46,47]. Nevertheless, a relatively high percentage of dogs included in this study did not show any clinical abnormality, as also previously shown [37]. False positive results may be due to the presence of larvae of cat lungworms in the feces of dogs [48], thus explaining the absence of clinical signs in dogs shedding larvae. Nonetheless, this is reasonably not the case of the present study, as all larvae were adequately examined microscopically, and feline metastrongyloid larvae have distinctive features that allow for their discrimination by expert operators [49].

The unpredictable nature of canine angiostrongylosis is hereby confirmed as having very highly variable clinical pictures. Although knowledge of clinical features has been greatly expanded in the last two decades, the full spectrum of clinical signs is yet to be completely elucidated, especially in the case of aberrant localizations of both adult and larval *A. vasorum*. Adult parasites have been found in the pericardial sac and in the urinary bladder of dogs [50], and intraocular localizations have also been reported [28]. Experimental studies have also shown that *A. vasorum* adults may migrate in the kidneys, causing renal cysts [51], and in the femoral arteries [52] or in the thoracic aorta [53], leading to their rupture and the subsequent death of infected animals. It has also been demonstrated that alive and active *A. vasorum* L1 can be retrieved in several organs and tissues other than blood and CNS, e.g., diaphragm, liver, pancreas and skin [50]. These ectopic localizations can cause unusual presentations, e.g., dermatitis [54], granulomatous hepatitis and multiple acquired portosystemic shunt [55], glomerulonephritis and granulomatous eosinophilic inflammation in the pancreas [56]. These data are still fragmentary and new studies aimed at exploring all possible clinical impacts of *A. vasorum* infection are advocated.

In conclusion, respiratory signs remain the predominant alteration in infected dogs even when more than one clinical alteration is found [4,18,20]. Nonetheless, the absence of cough or dyspnea should not prevent the inclusion of *A. vasorum* in the differential diagnosis for dogs with other compatible clinical signs, especially if living in enzootic areas. In fact, as shown here and in other studies [37], many infected dogs may display only non-respiratory signs or lack clinical signs entirely.

The presence of a high percentage of subclinical infections further underlines the primary importance of adequate routine parasitological examinations in the daily veterinary practice for all dogs living in endemic areas, as healthy dogs may present with a sudden onset of severe and life-threatening clinical angiostrongylosis [20,21,27,57]. Accordingly, the results presented here substantiate that *A. vasorum* may infect dogs more frequently than previously thought, despite the absence of compatible clinical signs, and suggest that, in the past, the lack of awareness and adequate routine parasitological tests may have led to an underestimation of subclinical infections.

## 4. Materials and Methods

**Figure 5:**
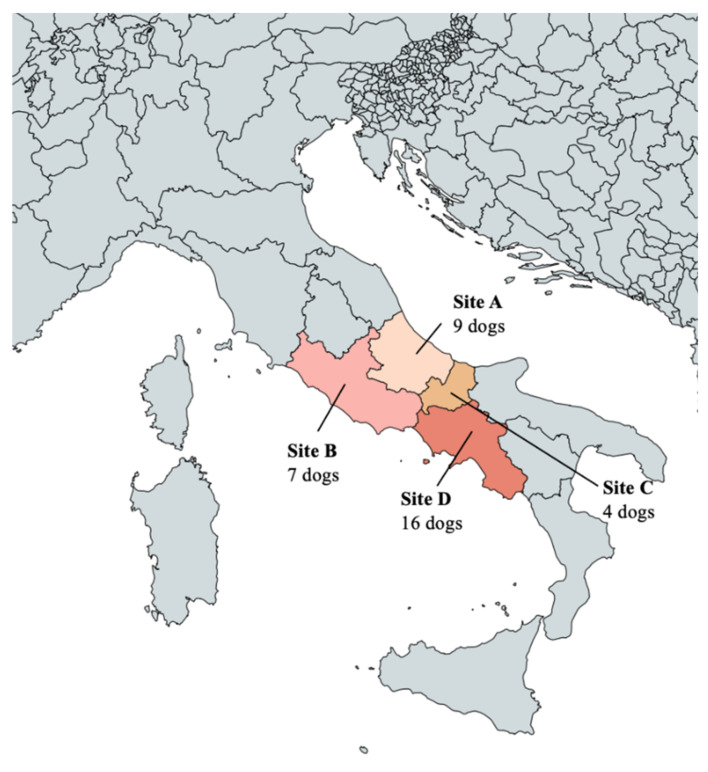
Map of Italy showing the study sites of the present study: A (Abruzzo); B (Latium); C (Molise); D (Campania).

Thirty-six privately owned dogs referred to veterinary facilities and practices in different regions of Italy for routine check-up or clinical examinations were included in the study. Overall, 9 dogs were from Abruzzo (Site A), 7 from Latium (Site B), 4 from Molise (Site C) and 16 from Campania (Site D) (Figure 5).

**Figure 6:**
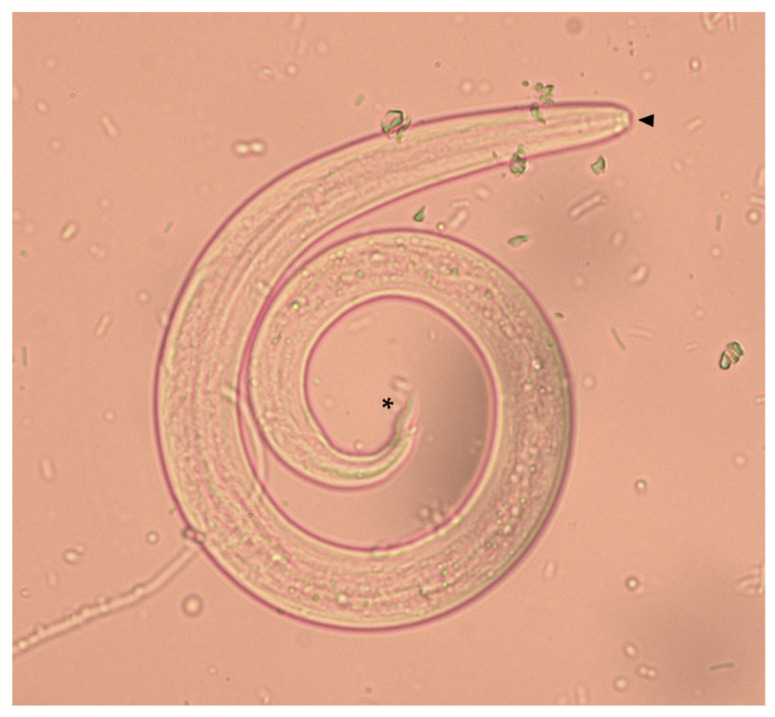
First stage larva (L1) of *Angiostrongylus vasorum* collected from an infected dog included in the present study. The anterior cephalic button (arrowhead) and the tip tail with the dorsal spine (asterisk) are indicated.

All dogs were subjected to a Baermann test performed by the examining veterinarian. All dogs scored positive for nematode L1 upon a Baermann test, and all larvae were identified as *A. vasorum* based on morphological and morphometric features [58,59]. In detail, *A. vasorum* L1 were identified based on their length (310–400 µm) and width (14–16 µm), on the presence of an anterior cephalic button and of a tip tail with a curve sinus wave and a dorsal spine (Figure 6) [9]. The 36 infected animals were clinically examined by the referring veterinarian, and a data sheet including signalment (age, sex and lifestyle) and presence of clinical signs was filled for each dog, none of whom was suffering from concomitant diseases.

## Figures and Tables

**Table 1:** Signalment of the dogs infected with *Angiostrongylus vasorum* involved in the study, along with presence (Y)/absence (N) of clinical signs.

Site	Dog ID	Age (Months)	Sex	Breed	Clinical Signs
Site A	16	120	Female	English Setter	Y
	58	96	Female	Mixed breed	N
	69	12	Female	Segugio italiano	Y
	70	24	Female	Mixed breed	Y
	74	4	Female	Segugio italiano	N
	75	5	Female	Labrador retriever	N
	82	96	Male	Mixed breed	N
	120	141	Male	Mixed breed	Y
	216	13	Female	Segugio italiano	Y
Site B	353	60	Female	Mixed breed	Y
	367	36	Female	Mixed breed	Y
	368	168	Female	Épagneul Breton	Y
	389	120	Male	Mixed breed	N
	413	48	Male	Segugio Maremmano	Y
	448	72	Female	Lagotto Romagnolo	Y
	455	12	Female	Cocker Spaniel	Y
Site C	694	24	Female	English Setter	Y
	710	24	Male	Mixed breed	Y
	723	36	Male	Labrador retriever	N
	746	24	Male	German Shepherd	Y
Site D	830	156	Male	Mixed breed	Y
	831	72	Female	German Shepherd	N
	832	120	Male	Mixed breed	N
	833	132	Male	Mixed breed	N
	834	24	Male	Mixed breed	N
	835	24	Male	Mixed breed	Y
	836	96	Male	Mixed breed	Y
	837	60	Male	Mixed breed	Y
	838	72	Female	English Setter	N
	839	36	Female	Mixed breed	Y
	840	24	Female	Mixed breed	Y
	841	24	Female	Epagneul Breton	Y
	842	36	Male	Lagotto Romagnolo	Y
	843	144	Female	Mixed breed	Y
	844	120	Male	Mixed breed	N
	845	120	Female	Mixed breed	N

**Table 2:** Number and percentages of clinical signs in 36 dogs of the present study infected by *Angiostrongylus vasorum*.

Category of Clinical Signs n/tot (%)	Clinical Sign	n/tot (%)
**Cardiorespiratory signs** 19/36 (52.8)	Cough	11 (30.5)
	Dyspnea	8 (22.2)
	Syncope	3 (8.3)
	Cyanosis	2 (5.6)
	Tachypnea	1 (2.8)
**Non-specific signs** 14/36 (38.9)	Weight loss	6 (16.7)
	Diarrhea	4 (11.1)
	Anorexia	4 (11.1)
	Lethargy	3 (8.3)
	Exercise intolerance	2 (5.6)
	Fever	1 (2.8)
**Signs related to coagulation disorders** 2/36 (5.6)	Hematochezia	1 (2.8)
	Hemorrhages	1 (2.8)
**Neurological signs** 1/36 (2.8)	Shivering	1 (2.8)
	Hypersalivation	1 (2.8)
**No clinical signs**		13 (36.1)

## Data Availability

All study data are presented in the article.
